# COVID-19 Emergence and Social and Health Determinants in Colorado: A Rapid Spatial Analysis

**DOI:** 10.3390/ijerph17113856

**Published:** 2020-05-29

**Authors:** Ivan J. Ramírez, Jieun Lee

**Affiliations:** 1 Department of Health and Behavioral Sciences, University of Colorado Denver, Denver, CO 80204, USA; 2 Consortium for Capacity Building/Institute for Arctic and Alpine Research, University of Colorado Boulder, Boulder, CO 80309, USA; 3 Department of Geography, GIS, and Sustainability, University of Northern Colorado, Greeley, CO 80639, USA; Jieun.Lee@unco.edu

**Keywords:** coronavirus, COVID-19, GIS, social determinants of health, multiple chronic conditions, spatial analysis, Colorado, pandemic

## Abstract

The aim of this rapid analysis was to investigate the spatial patterns of COVID-19 emergence across counties in Colorado. In the U.S. West, Colorado has the second highest number of cases and deaths, second only to California. Colorado is also reporting, like other states, that communities of color and low-income persons are disproportionately affected by COVID-19. Using GIS and correlation analysis, this study explored COVID-19 incidence and deaths from March 14 to April 8, 2020, with social determinants and chronic conditions. Preliminary results demonstrate that COVID-19 incidence intensified in mountain communities west of Denver and along the Urban Front Range, and evolved into new centers of risk in eastern Colorado. Overall, the greatest increase in COVID-19 incidence was in northern Colorado, i.e., Weld County, which reported the highest rates in the Urban Front Range. Social and health determinants associated with higher COVID-19-related deaths were population density and asthma, indicative of urban areas, and poverty and unemployment, suggestive of rural areas. Furthermore, a spatial overlap of high rates of chronic diseases with high rates of COVID-19 may suggest a broader syndemic health burden, where comorbidities intersect with inequality of social determinants of health.

## 1. Introduction

On March 11, 2020, the World Health Organization announced officially the coronavirus pandemic which was first detected in Wuhan, China in December of 2019 [1]. By March 16, 2020, the new coronavirus labeled COVID-19 had spread globally across 151 territories affecting 167,511 persons and killing more than 6500 [2]. Ten days later, the U.S. emerged as the epicenter of COVID-19, surpassing China’s case count [3,4]. As of April 13, 2020, the U.S. reported 582,468 cases of COVID-19 and 23,622 deaths [5], representing approximately 30% and 20% of the world’s COVID-19 morbidity and mortality. In the U.S., the state of Colorado, which is the focus of this study, reported the third highest number of COVID-19 cases (*n* = 7691) in the West, following the states of California and Washington, respectively [6,7]. Approximately 329 deaths were documented, since the first case of COVID-19 was reported on February 20 in Colorado [6,8].

Recent reports indicate, like in many other states [9], that COVID-19 incidence and deaths are disproportionately affecting communities of color in Colorado [10,11]. As of April 13, 2020, approximately 36.7% of cases and 26.7% of deaths were persons of Hispanic, non-Hispanic Black, and non-Hispanic Native Hawaiian or Pacific Islander origins, of which Hispanics shared the greatest burden of COVID-19 incidence and deaths [11]. Although the Colorado Department of Public Health and Environment (CDPHE) has yet to release socioeconomic information about COVID-19 cases, it is likely that these racial and ethnic disparities also correspond to inequalities in the social determinants of health, “the conditions in places where people live, learn, work, and play” [12]. As governmental and nongovernmental organizations report, communities of color and lower income persons face greater vulnerability to COVID-19 due to social, health and environmental disparities, such as lack of access to healthy foods, quality housing, health insurance and healthcare, and greater exposure to ambient air pollution [9,13]. Relatedly, such communities are also disproportionately affected by preexisting chronic conditions such as diabetes, cancer, and asthma [14], which may increase risk for severe COVID-19 health outcomes [15,16,17]. In Massachusetts, for example, researchers estimated that areas with higher levels of poverty, overcrowding, and populations of color were significantly associated with excess deaths during the first 15 weeks of 2020 [18]. Similarly, in New York, hospitalizations and deaths are distributed unevenly across racial and socioeconomic lines [19]. In Colorado, the Executive Director of CDPHE states, ‘We know that social and health care inequities affect outcomes, and that becomes even more apparent in times of disaster’ (as quoted in [10]).

Within this context, the aim of this rapid study was to examine the initial spatial patterns of COVID-19 in Colorado and explore preliminary associations with social determinants of health and chronic conditions. Using geographic information systems (GIS) and bivariate correlation analyses, this study investigated COVID-19 incidence and deaths from March 14, 2020 to April 8, 2020 at the county-level and the wider social, economic, and health context of emergence. Understanding the geographic patterns of COVID-19 in Colorado and the social determinants context may assist public health investigators with socially relevant insights, particularly as new information emerges about COVID-19 disparities among Colorado’s population.

## 2. Data

Publicly available COVID-19 data were downloaded daily from the Colorado COVID-19 online summaries, including geospatial information from CDPHE [6,20] and their Open Data Portal [21]. COVID incidence data include laboratory-confirmed and probable cases, and persons who tested positive for COVID-19 while visiting were assigned to the county where they were identified [22]. Social determinants of health data were retrieved from the social vulnerability index (SVI) dataset (2014–2018) from Centers for Disease Control and Prevention (CDC) (see [23] for variables’ details). These SVI data intended for the assessment of population vulnerability to disasters [24], include proxy variables for socioeconomic status, household composition, and minority status. To date, SVI data have been employed to examine wildfires [25], physical inactivity [26], and mental health and housing affordability [27]. Among SVI variables, a few indicators may be more relevant to vulnerability and exposure such as poverty and overcrowding, both of which have been correlated with influenza [28,29], and recently, COVID-19 [18], although at the census-tract and zipcode levels. Chronic conditions, such as asthma and diabetes hospitalizations (age-adjusted, per 100,000), heart disease mortality (age-adjusted, per 100,000), cancer mortality (age-adjusted, per 100,000), and obesity prevalence (adults, %), as well as influenza hospitalizations (age-adjusted, per 100,000) and mental health-related outcomes (suicide deaths and drug-related deaths, both per 100,000) were obtained from CDPHE [21], CDC [30], and County Health Rankings [31]. Health data represent the time period, 2013–2017, except for cancer (2012–2016) and obesity (2017). All datasets represent county-level estimates.

## 3. Methods

**Figure 1:**
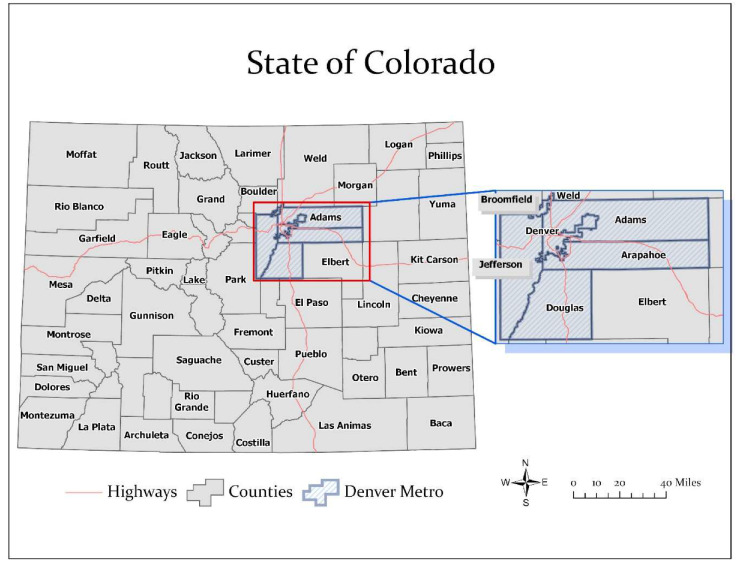
Map of Colorado counties, highlighting the Denver Metropolitan area. Source: CDPHE [21].

The state of Colorado has a population of 5,695,430 distributed across 64 counties, including those in the Denver metropolitan region (see Figure 1). Dot density and choropleth maps of COVID-19 cases for March 14, 2020 and April 8, 2020 were generated in ArcGIS Pro (Esri Inc., Redlands, CA, USA) [32]. An inverse distance weighted (IDW) algorithm was also used to interpolate and create a 3D continuous surface for mapping the sequential progression of COVID-19 rates across counties at five time points (March 14, March 18, March 25, April 1, and April 8). The fundamental assumption of IDW is that the interpolated surface is the result of locational dependency at the sampling points, whose influence decreases as the distance increases from its sampled location [33]. The IDW maps represent crude hotspots of COVID-19 incidence displayed from high (red) to low (blue) rates. To estimate the change in disease incidence, rate ratios comparing county-level COVID-19 incidence rate differences between March 14 and April 8 were calculated by dividing the current rate by the older rate. The ratio represents how many times higher the current rate is compared to the older rate. COVID-19-related mortality was assessed by calculating two estimates: percent of deaths in a county relative to the total number of deaths in Colorado and case fatality rate. The latter measurement was estimated by dividing county-level cases by county-level deaths and multiplying by 100.

COVID-19 outbreak data [34], which CDPHE began releasing on April 15, 2020 consisting of cases and deaths, associated with facilities by county, mainly for senior living, but also including meat-packing plants and jails, were also evaluated from March 11 to April 8.

To contextualize COVID-19 emergence, county-level profiles were generated to compare descriptively the wider social and health (i.e., chronic conditions, mainly) determinants context in Colorado. Rate ratios were calculated for each of the health outcome variables and social determinants of health indicators by dividing the county-level value by the state average. Similar profiles and rate ratios were utilized to contextualize mental health and housing disparities at the census tract-level in Colorado [27]. Potential associations between COVID-19 incidence and death rates with social and health determinants were estimated using Pearson’s correlation in IBM-SPSS (IBM Corp., Armonk, NY, USA) [35].

Lastly, using rate ratios described earlier, the spatial overlap of counties with several chronic conditions above the state average were compared with counties with higher rates of COVID-19 incidence.

## 4. Results and Discussion

### 4.1. Spatial Distribution of COVID-19 Incidence Rates

**Figure 2:**
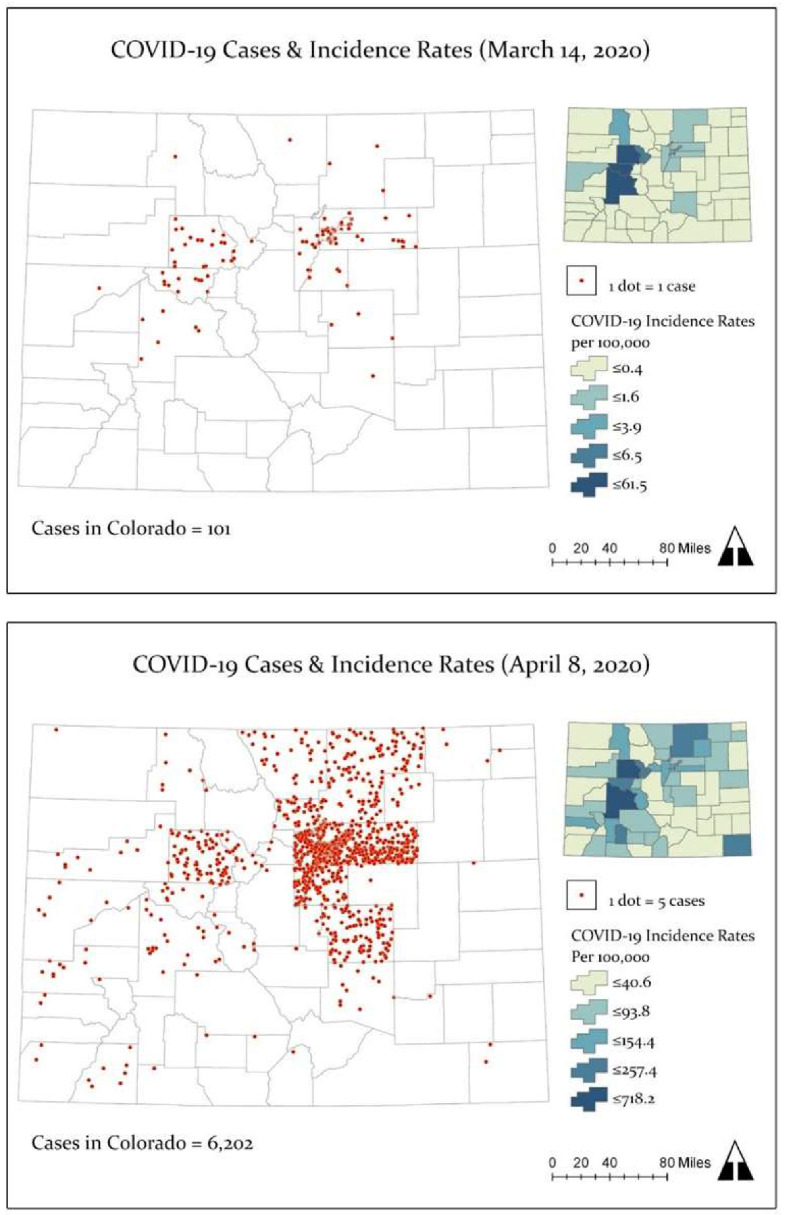
Maps of COVID-19 case counts (dot density) and incidence rates (per 100,000 persons) across counties in Colorado on March 14, 2020 (top panel) and April 8, 2020 (bottom panel). Source: CDPHE [6,20]

Figure 2 shows the spatial distribution of COVID-19 cases (dot density) and incidence rates for March 14, 2020 and April 8, 2020. On March 14, there were 101 cases of COVID-19 and 3 deaths distributed across 15 counties. The first cases reported in Colorado were mainly located in mountain communities in the Rocky Mountain West (e.g., Eagle, Gunnison, and Summit counties), the Denver metropolitan region (e.g., Arapahoe, Denver, Douglas, and Jefferson counties), the northern area of the Urban Front Range (Weld and Larimer), and El Paso County (see Figure 2 top panel). By April 8, Colorado reported a total of 6202 cases of COVID-19, 226 deaths, and 1221 hospitalizations. On April 8, the highest number of cases (*n* = 549) and deaths (*n* = 34) were documented. The state incidence rate was 108.9 (per 100,000 persons), the case fatality rate was 3.6%, and 19.7% of cases were hospitalized. As Figure 2 (bottom panel) illustrates, the number of counties that reported COVID-19 cases increased, from 15 to 56 out of 64 counties. The highest and lowest incidence rates (excluding counties without cases) were reported in Eagle County (718.2 per 100,000) and Prowers County (8.3 per 100,000).

### 4.2. Spatial Distribution of COVID-19 Incidence Rates

**Figure 3:**
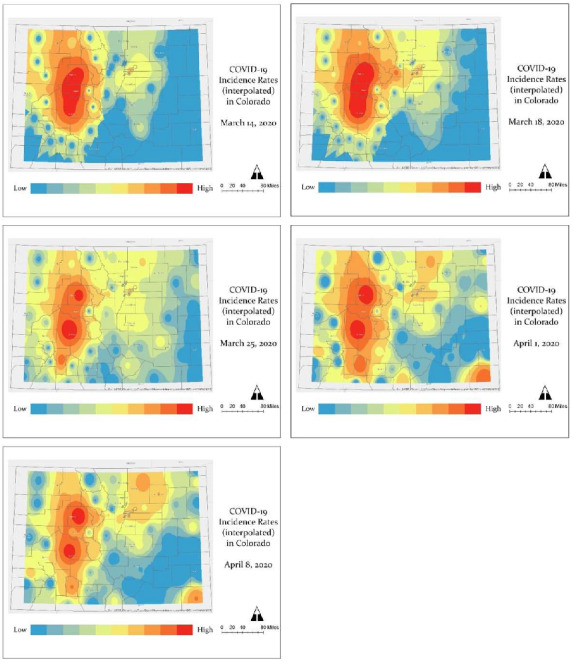
Maps of COVID-19 Incidence rates (per 100,000 persons) across five time points: March 14; March 18; March 25; April 1; and April 8, 2020, using the inverse distance weighted interpolation method. Incidence rates are displayed as high (red) to low (blue) intensity. Source: CDPHE [6,20].

Figure 3 displays the spatial progression of COVID-19 incidence across five time points: March 14, March 18, March 25, April 1, and April 8. Incidence rates are displayed from high (red) to low (blue) intensity. Initially, the centers of COVID-19 incidence were the mountain communities and ski towns, popular tourist destinations, west of Denver. By March 25, the spread of COVID-19 incidence expanded along the Urban Front Range, where centers of risk were quickly evolving from Denver and its metropolitan counties to El Paso (south of Denver) and Weld (north of Denver) counties. The geography of low risk (blue) diminished greatly across the state. On March 25, 36 counties were reporting cases of COVID-19, which then increased to 51 counties by April 1. Between April 1 and April 8, the intensity of COVID-19 risk around Denver and northern Colorado, e.g., Weld County, had vastly increased. There were also new possible centers of risk in southeastern Colorado (e.g., Baca), and counties north and south (e.g., Pitkin) of Gunnison, Eagle and Summit.

Table 1 shows the change in COVID-19 incidence rates between March 14 and April 8 expressed as ratios. The counties by rank order according to the greatest increase in disease incidence are displayed. The greatest positive change in COVID-19 incidence was observed in Weld County, where the rate increased by 204.7 times, followed by El Paso and Larimer counties, both where rates increased by approximately 174–178 times since March 14.

### 4.3. COVID-19-Related Deaths

Table 2 shows the counties with greatest percentage of deaths and their case fatality rates by April 8. Five counties (Denver, Weld, El Paso, Jefferson, and Arapahoe) accounted for 76.7% of all deaths in Colorado. Among these counties, Weld and El Paso had the highest case fatality rates. In general, the highest percentages of deaths were located along the Urban Front Range, whereas the highest case fatality rates were generally located in rural counties outside of the Urban Front Range (see Table A1 in Appendix A), with the exception of El Paso.

### 4.4. Spatial and Temporal Distribution of COVID-19 Outbreaks

**Figure 4:**
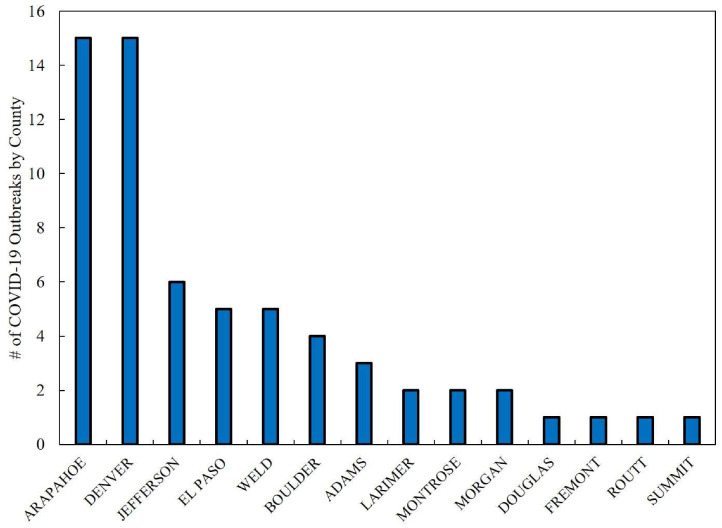
Number of COVID-19 outbreaks by county in Colorado. Outbreaks are associated with facilities such as seniors and assisted living, meat packing plants, and jails, from March 11 to April 8. Source: CDPHE [34].

Figure 4 displays the number of COVID-19 outbreaks associated with facilities, mainly for senior living, but also including meat packing plants and jails. A total of 63 outbreaks were reported from March 11 to April 8, 2020. Outbreak-related cases and deaths accounted for 12.9% and 55.3% of the total COVID-19-related morbidity and mortality in Colorado. The counties of Arapahoe and Denver reported the highest number of outbreaks (*n* = 30 in total). The highest number of COVID-19 cases and deaths associated with outbreaks were documented in Weld, Arapahoe, and Denver counties. Together these three counties represented an estimated 61.6% (*n* = 492) and 64.8% (*n* = 81) of all outbreak-related cases and deaths. Among these counties, Weld was the most affected with a reported 204 cases and 27 deaths.

Table 3 lists the number of COVID-19 outbreaks with cases and deaths sequentially from March 11 to April 8. As Table 3 indicates, beginning on March 23, COVID-19 outbreaks were reported every day, with the exception of March 26, until April 8. On April 3 the highest number of outbreaks, cases and deaths were documented. Approximately 40.0% (*n* = 102) of COVID-19 case counts on April 3 were linked to an outbreak at a meatpacking plant in Weld County [21].

### 4.5. COVID-19 and Social and Health Determinants Associations

Preliminary associations with social and health determinants and COVID incidence were assessed descriptively that highlight ratios comparing values of indicators generally with state averages. Table 4 shows profiles of the seven counties with the highest COVID-19 incidence rates in Colorado. In bold are ratios above 1 which generally represent the number of times higher a variable is compared to the state average for variables that suggest higher social vulnerability (the exception is the per capita income variable where a higher ratio indicates a higher socioeconomic status and therefore, suggests less social vulnerability). Some indicators that appear significant (e.g., by frequency) include limited English, single parent household, no health insurance, multiple unit structures, overcrowding, housing cost burden, and population density. Other indicators, although not as frequent suggest that COVID-19 incidence overlapped with higher rates of chronic conditions like asthma and diabetes, and also influenza, particularly in Denver, Morgan, and Weld counties.

Using Pearson’s correlation, the analysis showed that COVID-19 incidence rates were positively associated with per capita income (*r* = 0.32, *p*-value = 0.009) and multiple unit structures (*r* = 0.40, *p*-value = 0.001), and negatively associated with mobile homes (*r* = −0.31, *p*-value = 0.014). The aforesaid correlations, although not causative, suggest urban settings, relative to rural settings were at greater risk for COVID-19 incidence. Another correlation analysis focused on percentage of COVID-19 deaths and social and health determinants further supported an urban connection to incidence (see Table 5). In addition to per capita income and multiple unit structures, population density and asthma hospitalizations were significantly and positively associated with percentage of COVID-19 deaths, as well as minority, all variables that are characteristic of urban areas. Among these variables population density had the strongest association (*r* = 0.60; *p*-value = 0.000). CDC reports higher cumulative incidence of COVID-19 in urban areas with greater concentrations of people and interactions, which may facilitate a greater exposure to airborne transmission of the virus, e.g., in mass gatherings, events [36], and public transit [37].

Table 6 displays associations between social and health determinants and the case fatality rates of COVID-19. Unlike percentage of deaths and incidence, the case fatality rates of COVID-19 suggest that rural areas are at greater risk to death. Social determinants such as poverty and unemployment, variables which have higher prevalence in rural areas in Colorado, were significantly and positively associated with COVID-19 case fatality rates. Although many of the counties with the highest case fatality rates in Colorado are places with smaller populations, which can be misleading, these areas are least equipped to manage COVID-19 risk. For example, many rural counties depend on Medicaid (~38.7%) and several are without hospitals (~23.4%), according to the Colorado Health Institute [38]. In addition, internet disparities may affect rural populations’ access to COVID-19-related information for prevention and to seek care. Broadband access is not widely available in rural areas in Colorado and approximately 23.0% of the rural population does not have access to high-speed internet [39].

### 4.6. COVID-19 and Chronic Conditions

Table 7 compares rate ratios of COVID-19 incidence with an array of multiple chronic conditions in counties with higher incidence rates of COVID-19. Cancer and heart disease represent age adjusted mortality rates. As the table indicates, 31.6% (*n* = 6) of these counties had at least three out of five chronic conditions with rates above the state average. Half of the counties with multiple chronic conditions were located along the Urban Front Range, while the other half of counties were located in rural eastern Colorado.

Initial reports of COVID-19 risk show that individuals with pre-existing chronic conditions such as hypertension, asthma, diabetes, and cancer may increase the chances for severe illness and death [15,16,17]. In our county-level study, the preliminary findings suggest a potential association between asthma hospitalization and COVID-19 deaths (%) and potential spatial overlap with COVID-19 incidence at the population-level. Among counties with COVID-19 incidence rates above the state average, 42.1% (*n* = 8) of counties also had higher rates of asthma hospitalizations. According to National Jewish Health [40], it is possible that COVID-19 may have a greater impact on asthma sufferers, which may experience more severe symptoms. In New York City, 5.0% of COVID-19 patients were also comorbid with asthma [41], although this percentage, thus far, is lower than what researchers expected [42].

By far, diabetes has been the most frequent pre-existing chronic condition associated with disease severity of COVID-19 [15]. Although a significant association between diabetes and COVID-19 incidence or death was not found in our analysis, this present study did find that 36.8% of counties (*n* = 7) with higher incidence of COVID-19 also had higher rates of diabetes hospitalizations. Another chronic condition that frequently overlapped with higher incidence rates of COVID-19 in Colorado was cancer incidence (31.6%, *n* = 6 counties). Initial research shows that cancer patients that contracted COVID-19 appear to be more likely to experience multiple severe outcomes [17].

## 5. Conclusions

In summary, this rapid analysis examined the geographic patterns of COVID-19 emergence during an initial period of spatial progression across counties in Colorado. From March 14 to April 8, 2020, COVID-19 incidence intensified around the first areas of detection, i.e., mountain communities and ski towns west of Denver (e.g., Eagle and Gunnison) and north and south of Denver’s metropolitan area (e.g., Weld and El Paso), and evolved into new hot spots of risk north and south of Eagle County, and in eastern Colorado. Overall the greatest increase in COVID-19 incidence rates was observed in Weld County, which reported the highest incidence rate along the Urban Front Range, even higher than counties within Denver’s metropolitan area. Weld County also had the second highest percentage of deaths in Colorado, and the greatest number of cases and deaths associated with outbreaks at many senior living facilities and a meat packing plant, in particular, where 245 persons were infected of which 6 died [43].

A preliminary analysis of COVID-19 incidence and deaths reveals that percentage of deaths are higher along the Urban Front Range, while the rate of death (i.e., case fatality) is potentially higher in some rural counties with smaller populations. Some social and health determinant factors associated (not causal) with patterns of higher COVID-19-related death rates were population density and asthma hospitalization, suggestive of urban areas, and poverty and unemployment, suggestive of rural areas.

Although in general chronic conditions were not correlated with COVID-19 incidence, except asthma, an assessment of spatial overlap of multiple chronic conditions with COVID-19 suggests that areas of higher rates of several chronic conditions (e.g., diabetes and cancer) may potentially coincide (i.e., at the county-level) with areas of higher rates of COVID-19. In Colorado, the number of people diagnosed with multiple chronic conditions has been rising. In 2015, the prevalence of multiple chronic conditions (MCCs) among Colorado’s adult population, particularly older individuals—a growing demographic—was 35.2% [44], an estimate significantly higher than the U.S. average (25.7%). As chronic conditions accrue, they decrease the quality of life and increase risk for mortality [45]. For public health, this may suggest a higher number of individuals vulnerable to COVID-19 in certain counties, and draws attention to a larger syndemic health problem where synergies between COVID-19 intersect with MCCs, as well as inequality of social determinants of health.

As lockdown restrictions began to ease in Colorado in early May [46], as well as across the U.S., the state reported 16,635 confirmed and probable cases of COVID-19, 842 deaths, and 163 outbreaks at facilities, many senior living-related, across 56 counties [6]. Colorado at this time ranked second among states in the U.S. West in the number of cases and deaths [7]. As the number of COVID-19 cases continue to rise, racial and ethnic disparities in COVID-19 incidence increase as well. Approximately, 45.3% of cases are Hispanics and Blacks (as of May 3), even though these communities only make up 25.6% of Colorado’s population [6].

Although testing in Colorado has increased (as of May 3, 81,352 persons have been tested), the capacity to adequately address the pandemic in the state remains limited by supply (according to the Governor) [47]. Colorado’s COVID-19 response is also challenged by the ability of communities to decrease exposure to COVID-19 by social distancing, an important preventive measure for respiratory diseases. According to the Colorado Health Institute [48], which developed an index in Colorado based on overcrowding, population density, and workplace data (e.g., essential workers), the practice of social distancing is challenging for lower income persons and communities of color, including immigrants, because of crowded housing and work that requires they be present and work in close proximity to others. The Colorado Health Institute index suggests that social distancing is not only a problem for urban areas in the Denver metro, such as the suburbs of Adams County. It is also a challenge for rural communities within counties such as Weld, where there is a higher proportion of persons working in low-wage essential jobs [48] with greater exposures to COVID-19, and the least capacities to cope with health effects (e.g., with health insurance) and the economic fallout.

This study has several limitations given the nature of a rapid study (i.e., short time frame) and the rapidly evolving context of the pandemic, which includes changes to the counts of new cases and deaths as new information updates previous daily summaries posted online. The most recent assessment of case counts in Colorado shows a difference of 26 cases between the estimate used in this study for April 8 and May 3, for example [6]. That is approximately a 1% difference in cases. Additionally, testing is limited in Colorado, and information by county is not yet available publicly to gain a better understanding of how widespread COVID-19 was geographically. Furthermore, the study did not have access to geographic-level race/ethnicity or socioeconomic information about COVID-19 incidence. Therefore, evaluating the impact of social determinants on communities of color through correlations at the population-level is only a preliminary view. Lastly, the urban/rural differences (e.g., higher death rates and unemployment in rural areas) found in this study warrant further examination since we only analyzed county-level estimates. Conducting a finer analysis with more locally spatial information including socioeconomic and race/ethnicity data, for example, may reveal that within counties death rates are much higher in urban areas with high unemployment, poverty and other social disparities, as a recent study has shown in Massachusetts [18]. Nevertheless, our study provides potential insights for future investigators to consider when additional COVID-19 data becomes available, including census tract-level estimates and demographic information to better understand geographic patterns and social and health risk factors.

## Figures and Tables

**Table 1:** Estimated change in county-level incidence rates (IR) in Colorado between March 14, 2020 and April 8, 2020.

County	IR (Per 100,000)-3/14/20	IR (Per 100,000)-4/08/20	Change Ratio
WELD	1.0	195.4	204.7
EL PASO	0.4	74.8	178.0
LARIMER	0.3	49.7	174.0
ARAPAHOE	1.5	134.7	87.7
ADAMS	1.2	92.9	79.2
JEFFERSON	1.6	113.9	73.3
DOUGLAS	1.2	78.8	67.5
DENVER	2.9	143.6	49.1
PUEBLO	0.6	28.7	48.0
MESA	0.7	20.8	32.0
ROUTT	3.9	124.6	32.0
*BOULDER*	*2.2*	*66.7*	*31.0*
SUMMIT	6.5	184.0	28.5
EAGLE	36.5	718.2	19.7
GUNNISON	34.9	576.5	16.5

Italicized indicates since 3/18/20 Source: CDPHE [6,20].

**Table 2:** Top ten counties by percentage of deaths and case fatality rates in Colorado by April 8, 2020.

County	Deaths	Deaths (%)	Case Fatality Rate (%)
DENVER	38	16.8	3.7
WELD	36	15.9	5.9
EL PASO	32	14.2	6.0
JEFFERSON	25	11.1	3.8
ARAPAHOE	24	10.6	2.7
ADAMS	16	7.1	3.4
DOUGLAS	10	4.4	3.7
LARIMER	9	4.0	5.2
BOULDER	7	3.1	3.2
EAGLE	5	2.2	1.3

Source: CDPHE [20].

**Table 3:** Number of COVID-19 outbreaks with cases and deaths in Colorado from March 11, 2020 to April 8, 2020.

Date	COVID-19 Outbreaks	Cases	Deaths
3/11	1	4	0
3/12	1	24	1
3/20	1	24	4
3/23	1	10	5
3/24	2	54	13
3/25	1	2	0
3/27	1	50	17
3/28	1	9	3
3/29	1	10	1
3/30	5	76	12
3/31	1	9	2
4/1	7	85	15
4/2	4	28	4
4/3	10	252	23
4/4	1	5	2
4/5	5	22	7
4/6	6	56	7
4/7	7	39	4
4/8	7	40	5
Total	63	799	125

Source: CDPHE [34].

**Table 4:** Profiles of social and health determinants in seven high COVID-19 incidence rate Colorado counties.

Determinant	Denver	Eagle	Gunnison	Morgan	Pitkin	Summit	Weld
Health							
COVID-19 Incidence *	1.7	8.5	6.8	1.8	2.6	2.2	2.3
*COVID-19 Case Fatality* **	3.7	1.3	1.0	0.0	5.1	0.0	5.9
Asthma Hospitalizations *	1.6	0.6	0.5	1.3	0.5	0.5	1.5
Diabetes Hospitalizations *	1.5	0.4	0.4	1.7	0.2	0.3	1.4
Influenza Hospitalizations *	1.4	0.6	0.8	1.6	0.7	0.9	1.4
Drug-related Deaths *	1.6	0.5	0.0	0.7	0.0	0.7	0.8
Suicide Deaths *	0.7	0.6	1.4	0.7	0.9	0.9	0.8
Economic Stability							
Poverty **	1.1	0.5	1.0	0.8	0.6	0.8	0.8
Per Capita Income ****	1.5	1.5	1.1	0.9	2.0	1.4	1.1
Unemployment **	0.8	0.4	1.1	0.9	0.8	0.7	0.9
Education							
Limited English **	2.5	2.9	0.2	3.4	0.3	1.5	1.5
No High School Diploma **	1.4	1.2	0.3	2.2	0.4	0.7	1.3
Family/Social Support							
Single Parent Household **	1.1	0.8	1.1	1.7	1.2	0.7	1.2
Healthcare							
No Health Insurance **	1.1	1.5	1.1	0.8	1.4	1.9	0.8
Housing							
Multiple Unit Structures **	4.7	3.4	1.2	1.0	3.3	4.9	0.9
Overcrowding **	1.3	1.7	0.8	1.7	0.6	2.4	1.1
Housing Cost Burden **	1.2	1.1	1.3	0.7	1.2	1.5	1.0
Race/Ethnicity							
Minority **	1.8	1.3	0.5	1.6	0.6	0.7	1.3
Urban/Rural							
Population Density ***	4523.5	32.3	5.1	22.1	18.4	50.0	74.0

* per 100,000 persons; ** %; *** persons per square mile; **** US dollar; italicized indicates actual value, not ratio. Sources: CDPHE [6,20,21]; CDC [23].

**Table 5:** Correlations between COVID-19 deaths (%) and social and health determinants in Colorado.

Determinant	Correlation	*p*-Value
Pop Density (People Per Sq. Mile)	0.60	0.000
Per Capita Income (Us $)	0.27	0.031
Minority (%)	0.23	0.072
Multiple Unit Structures (%)	0.44	0.000
Mobile Home (%)	−0.42	0.001
Asthma Hospitalization Rate (Per 100,000)	0.38	0.002

Source: CDPHE [6,21]; CDC [23].

**Table 6:** Correlations between COVID-19 case fatality (%) and social and health determinants in Colorado.

Determinant	Correlation	*p*-Value
Population Density (People per sq. mile)	−0.01	0.921
Poverty (%)	0.33	0.009
Unemployment (%)	0.53	0.000
Per Capita Income (US $)	−0.18	0.162
Mobile Home (%)	0.21	0.103

Source: CDPHE [6,21]; CDC [23].

**Table 7:** Rate ratio (number of times above the state average) comparison of COVID-19 incidence rates and multiple chronic conditions in Colorado.

County	COVID-19	Asthma	Cancer	Diabetes	Heart Disease	Obesity	MCC
Adams *	1.1	1.6	1.2	1.5	1.0	1.2	4
Arapahoe *	1.6	1.5	1.0	1.2	0.9	1.0	2
Baca **	3.0	1.0	1.1	1.2	1.2	1.0	3
Chaffee	1.5	0.8	1.1	0.7	0.9	0.8	1
Clear Creek *	1.2	1.2	1.0	0.6	1.8	0.9	2
Denver *	1.7	1.6	1.1	1.5	1.0	0.8	3
Eagle	8.5	0.6	0.6	0.4	0.5	0.6	0
Gunnison	6.8	0.5	0.8	0.4	0.6	0.7	0
Hinsdale	1.5	0.3	0.0	0.2	0.6	0.9	0
Jefferson *	1.4	1.3	1.0	0.9	1.0	0.9	1
Mineral	3.1	0.5	0.0	0.5	0.9	0.8	0
Montrose	1.1	0.8	1.1	0.9	1.1	1.0	2
Morgan **	1.8	1.3	1.2	1.7	1.2	1.5	5
Phillips **	1.1	1.3	1.0	1.3	1.5	1.1	4
Pitkin	2.6	0.5	0.5	0.2	0.4	0.7	0
Routt	1.5	0.6	0.8	0.4	0.6	0.6	0
San Miguel	1.6	0.3	0.7	0.4	0.6	0.8	0
Summit	2.2	0.5	0.5	0.3	0.6	0.7	0
Weld *	2.3	1.5	1.0	1.4	1.0	1.2	3

* urban front range counties; ** rural eastern counties. Source: CDPHE [20,21], CDC [30], and County Health Rankings [31].

## Appendix A

**Table A1:** Top ten counties by case fatality rates (%) in Colorado.

County	Cases	Deaths	Case Fatality Rate (%)
CROWLEY	1	1	100.0
ALAMOSA	7	2	28.6
OURAY	4	1	25.0
DELTA	6	1	16.7
MONTEZUMA	8	1	12.5
CHAFFEE	26	3	11.5
ELBERT	10	1	10.0
TELLER	12	1	8.3
MONTROSE	38	3	7.9
EL PASO	534	32	6.0

Source: CDPHE [6,20].
